# Implementation of Coach McLungs^SM^ into primary care using a cluster randomized stepped wedge trial design

**DOI:** 10.1186/s12911-022-02030-1

**Published:** 2022-11-04

**Authors:** Thomas Ludden, Katherine O’Hare, Lindsay Shade, Kelly Reeves, Charity G. Patterson, Hazel Tapp

**Affiliations:** 1 Department of Family Medicine, Atrium Health, Charlotte, NC USA; 2 School of Health and Rehabilitation Sciences, University of Pittsburgh, Pittsburgh, PA USA

**Keywords:** Asthma, Shared decision making, Motivational interviewing, Health information technology

## Abstract

**Background:**

Asthma is a prevalent chronic disease that is difficult to manage and associated with marked disparities in outcomes. One promising approach to addressing disparities is shared decision making (SDM), a method by which the patient and provider cooperatively make a decision about asthma care. SDM is associated with improved outcomes for patients; however, time constraints and staff availability are noted implementation barriers. Use of health information technology (IT) solutions may facilitate the utilization of SDM. Coach McLungs^SM^ is a collaborative web-based application that involves pediatric patients, their caregivers, and providers in a personalized experience while gathering patient-reported data. Background logic provides decision support so both audiences can develop a well-informed treatment plan together. The goal of this study is to evaluate the implementation of the Coach McLungs^SM^ intervention into primary care.

**Methods:**

Implementation will be evaluated using a stepped wedge randomized control study design at 21 pediatric and family medicine practices within a large, integrated, nonprofit healthcare system. We will measure changes in emergency department visits, hospitalizations, and oral steroid use, which serve as surrogate measures for patient-centered asthma outcomes. We will use a generalized linear mixed models with logit link to test the hypothesis for the reduction in exacerbation rates specifying the fixed effects of intervention and time and random effects for practice and practice*time. This design achieves 84% power to detect the hypothesized effect size difference of 10% in overall exacerbation between control (40%) and intervention (30%) periods (two-sided, *p* = 0.05). Implementation will be guided using the Expert Recommendations for Implementing Change (ERIC), a compilation of implementation strategies, and evaluated using the CFIR (Consolidated Framework for Implementation Research) and RE-AIM (Reach Effectiveness, Adoption, Implementation, Maintenance).

**Discussion:**

We anticipate that a tailored implementation of Coach McLungs^SM^ across diverse primary care practices will lead to a decrease in emergency department visits, hospitalizations, and oral steroid use for patients in the intervention group as compared to the control condition.

*Trial Registration*: Clincaltrials.gov, NCT05059210. Registered 28 September 2021, https://www.clinicaltrials.gov/ct2/show/NCT05059210

**Supplementary Information:**

The online version contains supplementary material available at 10.1186/s12911-022-02030-1.

## Background

Asthma is a respiratory disease affecting the lungs which results in significant illness and death regardless of age. In the United States, asthma is one of the top chronic diseases affecting over 6 million children, with 80,000 hospitalizations, 185 deaths, over 500,000 Emergency Department (ED) visits, and more than 7.9 million missed school days every year [1–9]. Significant disparities in asthma outcomes occur by sex, race, ethnicity, and socioeconomic status [10–19]. Pediatric males have a higher prevalence of asthma and are more likely to have inpatient stays for asthma [14, 15, 20, 21]. Minority children are less likely to utilize controller medication [10, 11, 22–27] with African American children having 2 to 3 times higher rates of acute care visits compared with non-Hispanic white children and a 4.9-fold higher asthma death rate [10, 11, 28–31]. Inner cities, such as Charlotte, NC, are epicenters for asthma health disparities in the US, with children 10–17 years old bearing a disproportionate share of the burden [32–35].

Numerous individual- (provider, patient), interpersonal- (family), and system-level (healthcare policies, health systems operations) factors contribute to poor asthma outcomes [22, 36–42]. These documented disparities may persist due to the lack of a comprehensive approach for asthma care that is scalable, sustainable, and widely disseminated [22]. Previous programs, such as asthma action plans [43], allergen remediation [36, 37, 44–46], education [24, 47–49], peer support [50], case management [51–53], or community health workers [54–57], have shown positive results on a local level or in the short-term; however, no single program demonstrated widespread and sustainable outcomes improvement.

Improved asthma outcomes are associated with effective communication strategies between patients and providers [58–60]. Shared decision making (SDM) is a patient-centered process based on a platform of motivational interviewing, in which patients and providers work together to make decisions, select tests, treatments, and jointly create care plans based on evidence that balances risks with patient preferences and values. Previous studies show this SDM intervention is associated with improved outcomes for pediatric and adult patients with asthma [6, 61–67].

Clinical uptake of SDM has been slow [6, 68, 69] in part because of knowledge gaps on how to best implement and sustain complex interventions. Barriers, such as time constraints, limited support staff availability to serve as health coaches, lack of clinic resources, pressure on clinical sites to improve efficiency, and the complexity of electronic medical record (EMR) systems were noted implementation challenges in previous work. One example of a sustainability barrier is extra staff required to serve as coaches for a paper-based version of the asthma SDM tool [70–73]. While practice facilitation of complex interventions in primary care can show improved outcomes [74, 75], these approaches continue to show mixed results [76, 77].

Implementation challenges combined with rapidly advancing availability of new technologies, led to the development of a health information technology (IT) solution that removed known barriers to adoption and automated the health coach role, thus removing the need for a clinical staff member to serve as the health coach. Specifically, several key elements of SDM are now integrated into an collaborative on-line application called Coach McLungs^SM^.

This Coach McLungs^SM^ health IT solution virtually incorporates elements of in person SDM discussion using a conversational style through Coach McLungs^SM^ to (1) elicit patient asthma information, (2) provide personalized education, and (3) incorporate motivational interviewing [62]. Designed for the user to complete the experience prior to an asthma-specific clinic visit, the application offers patients and caregivers asthma education and opportunities for a more in-depth visit with their care providers. Use of this technology will extend healthcare professionals’ ability to deliver personalized care using an on-line health coach. In summary, Coach McLungs^SM^ integrates elements of the full facilitated approach into a SDM solution that is compatible with primary care practice workflows.

A paper version of Coach McLungs^SM^ was rigorously evaluated in previous studies [6, 61, 64]. This SDM intervention was associated with improved outcomes for both pediatrics and adults, such as reduced ED visits, hospitalizations, decreased rescue medication use, better adherence to asthma control medications, improved asthma-related quality of life, fewer asthma-related medical visits, better lung function, and improved perceptions of SDM [6, 61, 63, 64, 78]. The newly developed virtual version of Coach McLungs^SM^ SDM intervention is currently being evaluated in two pediatric EDs within separate healthcare systems [79].

Implementation research is required to best translate research findings into clinical care and enhance sustainability and ease of dissemination [80]. Implementation and dissemination was highlighted as a key national priority by the National Institutes of Health (NIH), Patient-Centered Outcomes Research Institute (PCORI), the Agency for Healthcare Research and Quality (AHRQ) and the Institute of Medicine (IOM) [81–83]. Yet, researchers are challenged to find reproducible implementation strategies. Implementation evaluation frameworks can offer consistent language and uncover identification of effective strategies across diverse healthcare settings. In recent years, multiple frameworks have been developed to provide guidance on how to evaluate implementation interventions (e.g. the Consolidated Framework for Implementation Research (CFIR) and Reach, Effectiveness, Adoption, Implementation, Maintenance (RE-AIM)) and even more recently, practical guidance and strategies on how to best organize implementation strategies have become available [80, 84, 85], such as the Expert Recommendations for Implementing Change (ERIC), a compilation of implementation strategies [80, 84, 85], that outline key implementation elements and evaluation strategies for use in primary care.

This study will evaluate the implementation of Coach McLungs^SM^ into primary care practices across a large healthcare system. We anticipate the implementation of Coach McLungs^SM^ into primary care will promote understanding of best practices for SDM adoption, improve outcomes for asthma patients, and through ease of use, will be sustainable and facilitate further dissemination of SDM interventions into other primary care settings both locally and nationally. End-users of this intervention will be patients, parents (or caregivers), providers, and practice staff. We anticipate a demonstration of efficacy will improve patient outcomes, particularly for the most at-risk patients. Ultimately, this knowledge may invite appropriate policy changes, such as providing insurance coverage and/or provider reimbursement for SDM, towards further use of SDM interventions.

## Aims and anticipated outcomes

Specific Aim 1: Primary care implementation and evaluation of *Coach McLungs*^*SM*^

Sub Aim 1.1: Convene the Stakeholder Advisory Committee (SAC).

The SAC will consist of researchers, providers, patients, local and national advocacy groups, and other stakeholders as previously described. The SAC will meet to oversee all aspects of the study from inception to dissemination.

Sub Aim 1.2: Implement *Coach McLungs*^*SM*^ at 21 primary care sites within a large healthcare system.

Before randomization, 21 primary care practices selected from 74 eligible practices for *Coach McLungs*^*SM*^ implementation must meet a minimum required number of asthma patients seen within the previous 18 months. Based on our previous experience implementing interventions in primary care practices and the flexibility of the 9-month window for implementation kick-off, we anticipate successful recruitment of all 21 practices within 9 months. After randomization of the 21 practices to the 5 steps of a stepped-wedge study design, we will contact the practice manager at each site to initiate a time for study kick-off lunch-and-learn training. Pre-implementation CFIR and Organizational Readiness for Implementation Change evaluation surveys will take place at study kick-off (see Additional files 1, 2). Practices will be given an iPad for patients to use *Coach McLungs*^*SM*^ in the waiting room or exam rooms prior to their asthma visits. The initial coach session takes 12–15 min to complete, and *Coach McLungs*^*SM*^ will save a personalized summary in the patient’s EMR. *Coach McLungs*^*SM*^ virtual character is adapted into this interactive, virtual form for children aged 5–17 and their caregivers. Preliminary testing with patients and providers across selected primary care practices ensured *Coach McLungs*^*SM*^ adopted appropriate health literacy, technical literacy, and social context [62].

Sub Aim 1.3: Evaluation of the barriers and facilitators to *Coach McLungs*^*SM*^ adoption at the practice and healthcare system level.

Additional provider CFIR evaluations will take place 6-, 12-, and 18-months post-implementation (see Additional files 3–5). Key informant interviews will be conducted at 6-months post intervention (see Additional files 6, 7).

Sub Aim 1.4: Conduct iterative process improvement through ongoing evaluation.

All outcomes and survey data will be collected and provided quarterly to the SAC in order to evaluate the implementation. Barriers and facilitators that emerge from 6-, 12-, and 18- month CFIR post-implementation surveys (see Additional files 3–5) will be reviewed by the SAC and feedback will be provided to the practices at 6-month follow up meetings.

Specific Aim 2: Conduct ongoing evaluation of patient-centered outcomes.

Based on a review of the implementation evaluation literature [61, 86, 87], the RE-AIM framework (Reach, Effectiveness, Adoption, Implementation, Maintenance) will be used for implementation evaluation. Reach, Maintenance: the number of patients and providers utilizing Coach McLungs^SM^ over time at each practice will be measured. As Coach McLungs^SM^ will be accessed through the EMR, we will be able to measure the number of times the intervention is delivered. Effectiveness: Patient health outcomes data of acute care visits for asthma exacerbations will be analyzed for patients who have a diagnosis of asthma (ICD-10 codes J45.0-J45.998) irrespective of intervention, allowing measurement of any outcome improvements over the life of the study. Data will be assessed at 6, 12 and 18 months for patient outcomes.

**Fig. 1:**
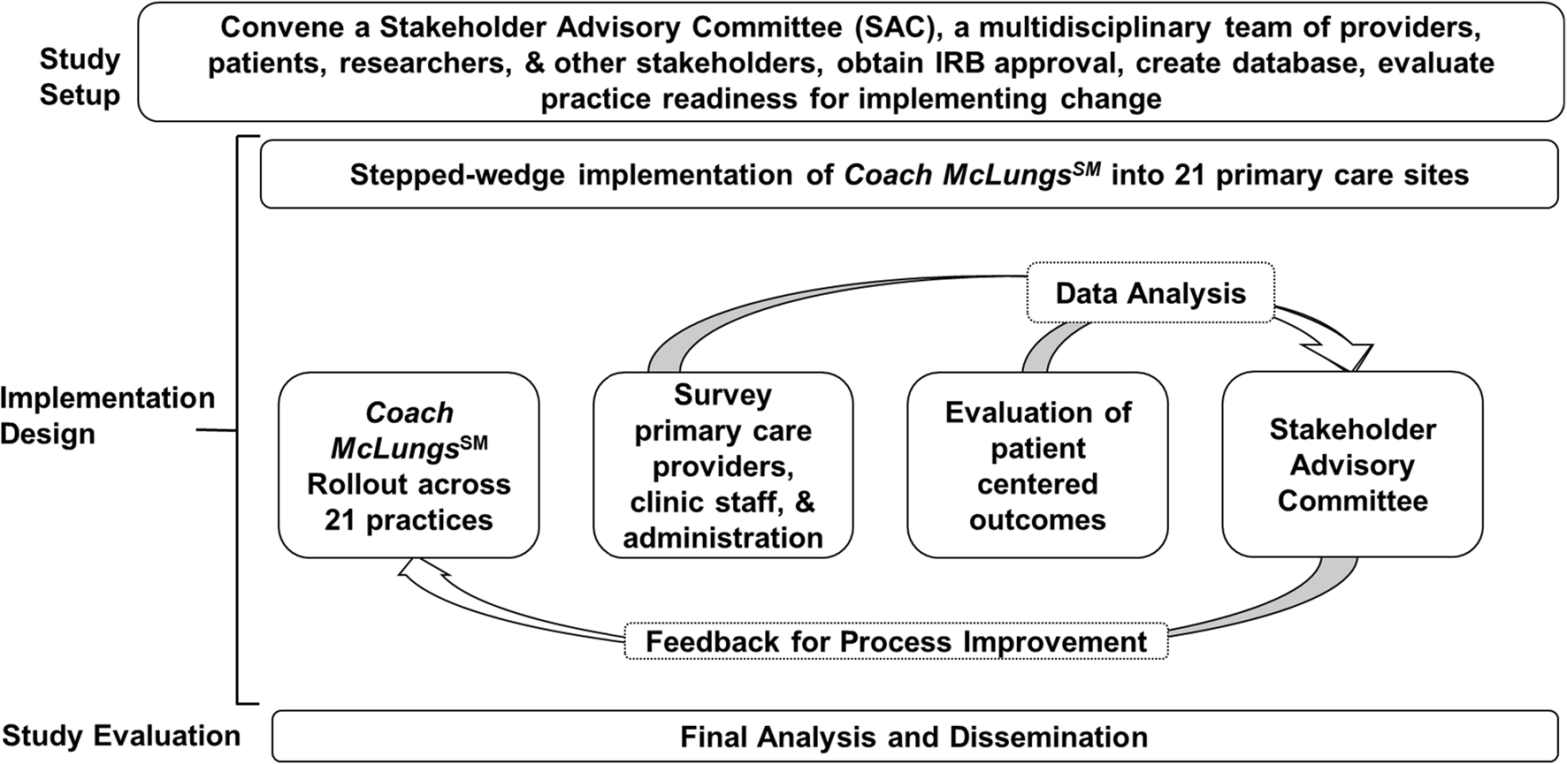
Study overview

Specific Aim 3: Dissemination: Disseminate results and strategies for primary care implementation both locally and nationally. We will disseminate through local stakeholders, practice-based research networks, asthma advocacy national organizations, and academic research meetings for healthcare, primary care and asthma. We expect multiple methods, qualitative and quantitative results manuscripts in subsequent years (Fig. 1).

## Methods

We propose cross-sectional stepped-wedge cluster randomized design with a phased, randomized rollout. Twenty-one practices will be assigned the intervention randomly over the course of 30 months with the first 6 months used as a baseline period (total rollout time period 36 months) [88]. The research team will be blinded to practices that will receive the intervention until 3 months prior to implementation. Randomization is generated by the study statistician using SAS version 9.4, stratified by practice size (Table 1). Once the intervention has been rolled out at the practice, patients will be exposed to Coach McLungs^SM^ as they are seen by providers for their asthma. This implies that crossover to the intervention is not only at the practice level but also potentially the patient level [89]. The patient level exacerbation rates will be measured at 6-, 12-, and 18-month intervals pre- and post- the interaction with Coach McLungs^SM^.

**Table 1:** Stepped-Wedge Design for *Coach McLungs*^*SM*^ Implementation with 6-month time intervals for each

	**Baseline (6 mos)**	*Time 1 (6 mos)*	*Time 2 (6 mos)*	*Time 3 (6 mos)*	*Time 4 (6 mos)*	*Time 5 (6 mos)*
Practices 1–4	**120**	*120*	*120*	*120*	*120*	*120*
Practices 5–8	**120**	**120**	*120*	*120*	*120*	*120*
Practices 9–13	**150**	**150**	**150**	*150*	*150*	*150*
Practices 14–17	**120**	**120**	**120**	**120**	*120*	*120*
Practices 18–21	**120**	**120**	**120**	**120**	**120**	*120*

Bold: control period, Italics: intervention period

The aim of implementation is to disseminate Coach McLungs^SM^ to patients most at need in the primary care setting. Often these patients have problems with managing asthma as a chronic disease and adhering to treatment plans. Primary care providers and staff are aware of the need to better prepare patients for chronic asthma management and fully support the Coach McLungs^SM^ SDM intervention.

Inclusion Criteria for this study are: (1) having attained the age of at least 5 years but less than 18 years at enrollment; (2) having established mild, moderate, or severe persistent asthma with a diagnosis verified by practice providers; (3) English or Spanish speaking. 4,981 patients in 21 practices aged 5–17 were seen in primary care for asthma in last 18 months (Table 2).

**Table 2:** Number of Primary Care Patients Age 5–17 Diagnosed with Asthma October 2017 to March 2019

	# Eligible patients at proposed 21 practices	# Patients in healthcare system
Aged 5–17	4981	14,283
Male	2985	8528
Female	1996	5755
African American	1995	4863
Caucasian	2364	7147
Other/Unknown	622	2273
Hispanic/Latino	313	1141
Non-Hispanic Latino	4080	10,784
Unknown Ethnicity	588	2358

Providers and clinic staff will offer Coach McLungs^SM^ to all patients and those deemed in need of extra asthma education. To simplify the selection process, practices may choose to offer Coach McLungs^SM^ to all patients with asthma, regardless of asthma severity. An additional enrollment table will track providers who are using Coach McLungs^SM^ at the participating practices. Patient-centered outcomes will be monitored from available EMR data throughout the course of the study by the research team. End-users will be primary care patients, providers, and staff. The types of patients will be primary care pediatric patients and their caregivers (most likely parents) with asthma, aged 5–17 (Table 2).

**Fig. 2:**
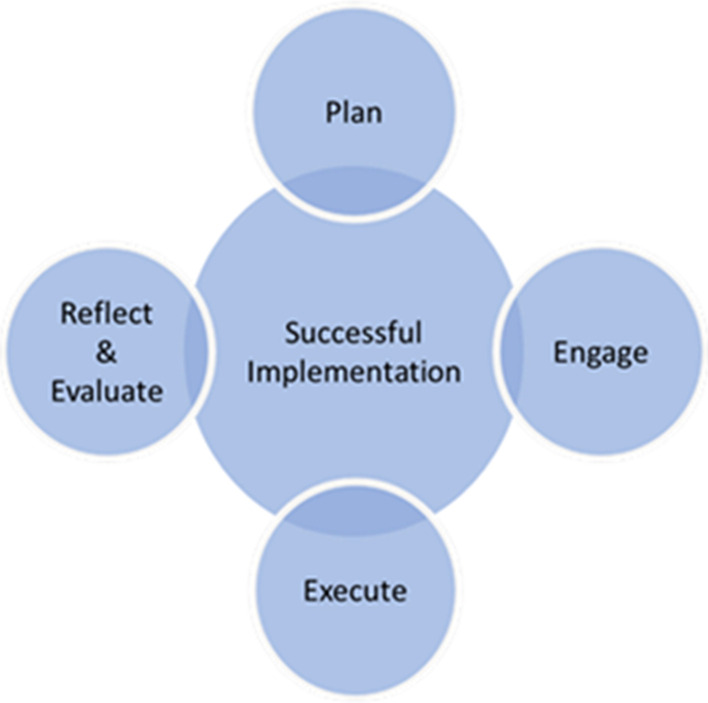
Process improvement

A key feature of our implementation approach will be the use of iterative process improvement cycles (Fig. 2) to implement this process which will be monitored by the SAC, to evaluate both the process and outcomes of the intervention.

Multiple patients, primary care providers, and staff engaged with the SAC through development of this virtual SDM intervention, a previous pilot study, and planning for this current implementation study in primary care are involved in process improvement. We plan to use an iterative quality improvement method, where the implementation will be adjusted at each practice based on feedback from the SAC after advisory members are updated with ongoing outcomes and survey results.

**Fig. 3:**
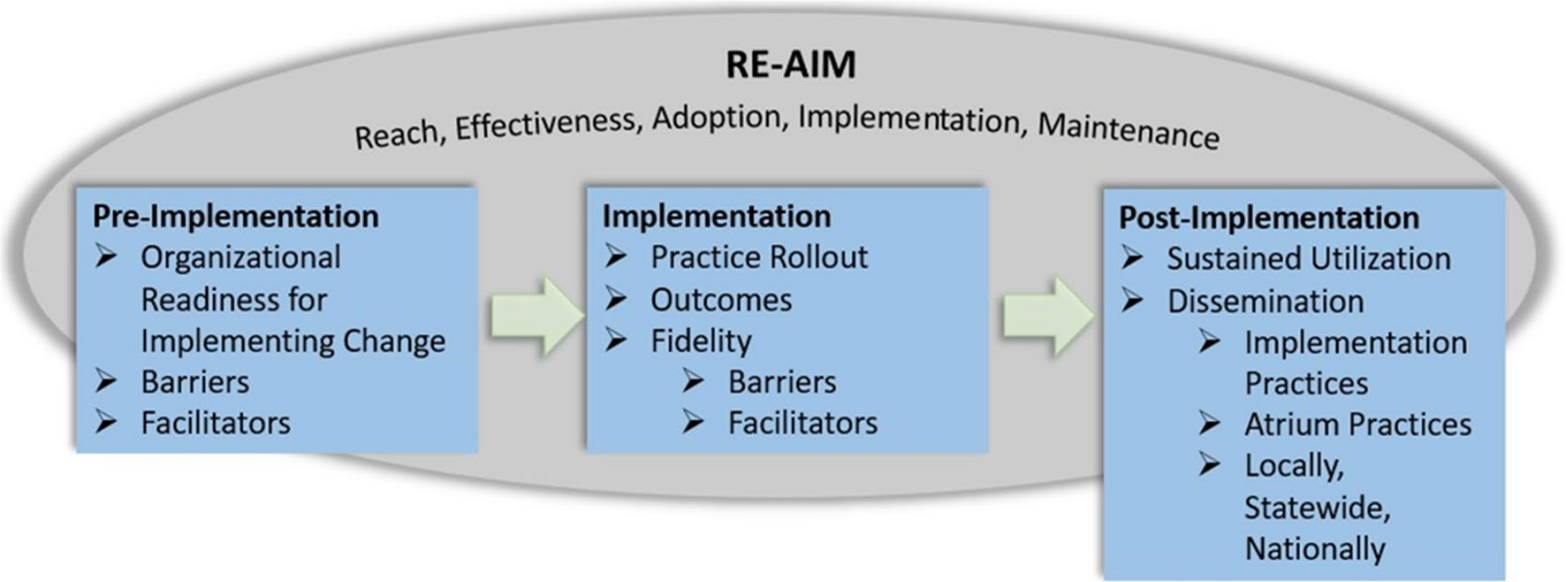
Conceptual model

Domains from the CFIR [90–94], such as inner setting (structural characteristics, culture, networks and communications, and tension for change) and outer setting (patient needs and resources, external policies, and incentives), will be incorporated into the implementation evaluation surveys (see Additional files 1, 3–5) [95–97]. The RE-AIM framework represents a flexible, comprehensive model according to which we will execute and evaluate the SDM implementation (Fig. 3). Adapting RE-AIM elements allows for full evaluation of the intervention at different time points during the study [98, 99].

Results from the RE-AIM-guided evaluation at baseline, during, and after the intervention will use outcomes data and participant input to inform implementation reach and assess barriers and facilitators, allowing for process adjustments (Table 3). CFIR survey measures and the Organizational Readiness for Implementing Change (ORIC) measure [95, 96, 100] will be used to assess collective perceptions about factors expected to affect implementation of Coach McLungs^SM^ (see Additional files 1–5).

**Table 3:** RE-AIM evaluation tools and measures by specific aim

Specific Aim 1: Primary care implementation and evaluation of *Coach McLungs*^*SM*^
Sub Aim 1.2 Implement the *Coach McLungs*^*SM*^ SDM intervention into 21 primary care practices
Sub Aim 1.3 Evaluate barriers and facilitators to implementation at practice and system level

Sub Aim	RE-AIM	Measures	Evaluation tools	# of months per practice			
				0	6	12	18
1.2	Reach and Implementation	Providers and Administration	CFIR / ORIC Survey (Pre-Implementation) 21 Practices	×			
1.3	Adoption and Implementation	Providers, Staff, Patients	Key Informant Interview 3 Practices		×		
		Providers	SDM-Q-Doc Survey 3 Practices			×	
		Providers	CFIR Survey (Post-Implementation) 3 Practices		×	×	×

Specific Aim 2: Conduct on-going evaluation of patient-centered outcomes						
RE-AIM	Measures	Evaluation tools	# of months per practice			
			0	6	12	18
Reach and Maintenance	Patients	*Coach McLungs*^*SM*^ use compared to the total # of eligible patients		×	×	×
Adoption	Patients	*Coach McLungs*^*SM*^ Completion %		×	×	×
Effectiveness	Health Utilization Outcomes	ED Visits, Hospitalizations, Oral Steroids, Exacerbations, and PCP Utilization	×	×	×	×
Adoption (Built-in Surveys)	Knowledge, Satisfaction, and Self-Efficacy	Patient Surveys		×	×	×
Reach and Maintenance	Providers	# of Providers using *Coach McLungs*^*SM*^		×	×	×

Reach will be considered the recruitment and retention of practices and providers in the study and the number of patients using Coach McLungs^SM^. Reach will also be the number of practices receiving the training for Coach McLungs^SM^ at kick-off, and the number and proportion of patient and providers at each practice receiving the intervention. Effectiveness will be based on health utilization outcomes. Patient utilization data of acute care visits for asthma exacerbations will be analyzed. Adoption will be based on the acceptability, barriers and facilitators to successful adoption of the intervention among participants. For patients’ adoption will be considered the number of patients who complete use of Coach McLungs^SM^ and the number of optional shared decision making knowledge, attitude, and self-efficacy asthma surveys built-in to Coach McLungs^SM^ [101]. The patient/caregiver user is prompted to complete the asthma surveys immediately after each initial coaching visit (see Additional file 8). For providers, we will use the validated 9-item questionnaire (SDM-Q-DOC) (see Additional file 9), and CFIR implementation evaluation surveys (see Additional file 1, 3–5), and key informant interviews (see Additional file 7), to determine the acceptability, barriers and facilitators to successful adoption from physicians’ perspectives [102, 103]. Implementation will be evaluated using ORIC surveys to assess practice readiness to implement change (see Additional file 2). Provider CFIR surveys will take place pre-implementation and post-implementation (see Additional files 1, 3–5). Maintenance number of recorded uses of Coach McLungs^SM^ will evaluate sustainability.

Anticipated barriers to Reach are based on our prior implementation knowledge of the need for practices to have flexibility in their approach to implementation. By using a tailored approach [99], we anticipate to fully and effectively implement Coach McLungs^SM^ at all 21 practices. We will (1) use a flexible approach with practices that includes a 6-month step time window in order to mitigate circumstantial barriers such as practice availability for kick-off; (2) address staff and provider preferences for each practice, such as who is needed at meetings, does the practice want a staggered or practice-wide approach to implementation; (3) the practice may request extra support for items such as extra training for communication skills (usually motivational interviewing or shared decision making), use of asthma guidelines, asthma medication management education; (4) practices may request support in considering how to adapt flow and timing of patient visits in order to maximize use of Coach McLungs^SM^. The implementation training will be adapted from the ERIC (Table 4).

**Table 4:** Implementation plan based on expert recommendations for implementing change (ERIC)

ERIC element	Element description for *Coach McLungs*^*SM*^ implementation
Use data experts	Director of data analytics will lead team to acquire, manage, and report data generated by implementation effort
Provide local technical assistance	Practice rollouts will be conducted by the lead research team with the implementation team providing technical assistance
Audit and provide feedback	Develop individual primary care practice feedback summaries including CFIR results and combined performance at other implementation and control sites and report out to practices at 6- 12- and 18- months post-implementation based on their preferences which include but no limited to: email, webinars, lunch-and-learn meetings and Skype conference calls
Use an implementation advisor	An implementation team will advise on the use of implementation frameworks and evaluation plan
Implementation facilitation	The lead reach team are both experienced practice facilitators with both *Coach McLungs*^*SM*^ and the previous paper-based asthma SDM toolkit
Assess for readiness and identify barriers and facilitators	Organization readiness assessment will take place at the system and practice level using the ORIC tool and elements from CFIR. Practice leadership, providers and patients will be surveyed and/or interviewed to evaluate barriers and facilitators
Develop an implementation blueprint	Manual of operation procedures will be developed to guide implementation strategies and goals
Organize implementation teams	The lead reach team will organize the implementation teams
Conduct educational outreach and ongoing training	Rollouts include kick-off lunch-and-learns consisting of 45-min training in patient/provider SDM communication, asthma guidelines and inhaler use refresher, and the use of *Coach McLungs*^*SM*^. Additional education training is based on practice needs and interest
Conduct educational meetings	The SAC will meet regularly, and other educational sessions will be targeted towards multiple stakeholders e.g. providers, practices adopting *Coach McLungs*^*SM*^, administration, community members, patients/caregivers
Engage community resources	Connect practices and patients to community resources with community partners

### Consent process

Since participant interviews are voluntary and will be anonymous, a waiver of documentation will be submitted to the IRB requesting that documentation of consent/assent will not be required. All staff involved in recruitment and retention will be required to successfully complete Human Subjects training and Health Insurance Portability and Accountability Act (HIPAA) training prior to working on this project.

Consent/assent forms describing in detail the study intervention, study procedures, and risks will be given to the participant. The following consent/assent materials are submitted with this protocol:

- Patient Informational Leaflet for Key Informant Interview.
- Provider Informational Leaflet for Key Informant Interview.

A verbal explanation will be provided in terms suited to the participant’s comprehension of the purposes, procedures, and potential risks of the study and of their rights as research participants. Participants will have the opportunity to carefully review the consent/assent form and ask questions prior to providing consent/assent for the study. Participants must be informed that participation is voluntary and that they may withdraw from the study at any time, without prejudice. A copy of the consent/assent document will be given to the participants for their records. The consent/assent process will be conducted and documented in the source document (including the date) before the participant undergoes any study-specific procedures. The rights and welfare of the participants will be protected by emphasizing the quality of their medical care will not be adversely affected if they decline to participate in this study.

### Study design

The stepped-wedge design is useful for the evaluation of complex healthcare interventions [89], particularly when the intervention is believed to be beneficial with minimal risk [104]. This design is increasingly being used to evaluate interventions involving healthcare delivery due to: (1) allowing the research team and clinical teams to rollout the intervention in small number of facilities in a timely, systematic manner [105]; (2) increasing participation and buy-in since all facilities will eventually get to implement the intervention during the study [89, 106, 107]; and (3) increase in statistical power compared to a cluster randomized trial due to increase in data collection and within cluster comparisons [89]. Our intervention is applied at the practice (cluster) level but the exacerbation rates are obtained on patients. In this cross-sectional cohort design, we will include both patients who are present at baseline and new patients coming into the practice throughout the study period. All patients in a practice would be eligible but providers may decide that some patients do not need to utilize Coach McLungs^SM^ [108].

### Sample size justification

We used a method to estimate sample size that takes into account the cluster randomization and the stepped-wedge design [88]. Our study design includes 21 clusters (facilities) with 6 time periods (including the baseline), 5 steps, 4 facilities switching from control to intervention at each step (except the third step will have 5), and an average of 30 patients per practice being continuously followed. Thirty patients is conservative as the typical number in a 6-month period is approximately 100. We conducted sample size analyses accounting for intracluster correlation (ρ = 0.20), individual autocorrelation (τ = 0.5), and cluster autocorrelation (π = 0.9) [88]. This design achieves 84% power to detect the hypothesized effect size difference of 10% in overall exacerbation between control (40%) and intervention (30%) periods (two-sided, α = 0.05). An effect size of 0.1 translates to a difference between groups of 10% absolute difference between intervention and control overall exacerbation rates.

### Analysis of outcomes

We will use a generalized linear mixed models with logit link to test the hypothesis for the reduction in exacerbation rates specifying the fixed effects of intervention and time and random effects for practice and practice*time. These random effects are based on the statistical model that accounts for separate correlations among individuals in the same cluster at the same or different time points, and random variation in the secular trends across clusters [89]. To control for repeated measures within individuals, we will include a separate random effect for each patient. We will test the effect of implementing Coach McLungs^SM^ with the intervention fixed effect (two-sided, *p* = 0.05) as well as mean difference and corresponding 95% confidence interval for the intervention effect. The study design and models proposed account for the establishment of causal treatment effects at the patient level [109].

### Data and safety monitoring plan

A Data and Safety Monitoring Plan (DSMP) will be implemented to ensure integrity of the study data and the safety of participants in the study. The plan will include the groups that are responsible for monitoring, the frequency for monitoring, and the information that will be monitored. In addition, the plan will define and describe the process for handling protocol deviations. All NHLBI data and safety monitoring guidelines will be followed. The principal investigator will be responsible for the oversight of data and safety monitoring. All Research Assistants responsible for data collection will be trained by the lead research team. All Research Coordinators must be certified as proficient in all study procedures prior to collection of data.

In order to track patient data linked to Coach McLungs^SM^, individual’s electronic medical record (EMR) numbers will be linked to study ID numbers. Names of participants with linkage to the ID number will be kept in a password-protected file on our secure server; only study staff will have knowledge of the password. Furthermore, this information, any consent forms and other forms with identifying information will be kept in locked filing cabinets that are separate from storage of de-identified data files. Only the research team will have knowledge of both the de-identified and identifiable data. Backup copies of all electronic data files will be updated immediately following the entry of new data. The backed-up version of the data file will be kept in a separate location on the server to protect against loss or damage. Furthermore, the network server upon which data files will be stored is backed up nightly to provide additional protection against loss. The lead research team will conduct error checking and preliminary analyses of all data to ensure accuracy prior to formal data analysis.

There are no physical risks anticipated from participation in this study. Since routine data are collected from the EMR, the participant burden will be low. Participants may feel concerned that their regular care will be impacted based on their decision to participate or not participate. Potential participants will be assured that their care will not be impacted by their decision to participate or not to participate in the study, and that they may withdraw at any time with no penalty. Data collected in the study will not be shared with anyone outside of the research team. Any signed consent and assent documents will be stored in a separate locked filing cabinet from identifiers. Only authorized staff will be allowed to access physical or electronic records from the study. Passwords will be used to protect computers and electronic files used for the project. Any breach of confidentiality will be immediately reported to the IRB.

### Groups responsible for monitoring

The protocol, informed consent and assent documents, and other relevant materials (such as surveys) will be reviewed and approved by the Atrium Health IRB. The IRB will be responsible for providing ethical oversight throughout the duration of the study, data analysis, and dissemination. Protocol deviations will be reported to the IRB according to IRB guidelines and the DSMP. All members of the study team will be involved in procedures to ensure subject safety and data integrity. All study personnel will receive training in human subjects’ protection and HIPAA, as well as study specific training on the protocol, procedures for maintaining data integrity and subject safety, applicable IRB policies, and on maintaining confidentiality of records.

Given the minimal risk associated with the intervention, we will utilize a data safety and monitoring board (DSMB) comprised of the lead research team. We will add one external non-study board member. The DSMB will meet annually to assess progress, preliminary data, and adverse events. Recommendations regarding the progress of the study will be documented for the study team, and potential concerns regarding the conduct of the study will be reported to the IRB or the Office of Clinical and Translational Research at Atrium Health.

### Monitoring study progress and subject safety

The study team will meet weekly or biweekly to review the progress of the study and any safety concerns. Information to be reviewed will include: (1) changes to the study protocol; (2) screening and enrollment log; (3) protocol violations; (4) reports submitted to the IRB. More frequent meetings may be required to address participant safety concerns. All reporting of protocol deviations will occur according to IRB requirements.

## Discussion

### Anticipated outcomes

All practices (100%) will receive a lunch-and-learn training on SDM communication and implementation of Coach McLungs^SM^. We assume 70% of patients and providers will begin adopting the intervention Coach McLungs^SM^. We expect that 50% of patients and providers patients will still be using Coach McLungs^SM^ by the end of the study.

Exacerbation outcomes will be measured over 18 months consisting of: ED visits, hospitalizations, and oral steroid prescriptions. We anticipate reductions in the proportion of intervention asthma patients with ED visits from 10 to 8%, patients with hospitalizations from 1.1% to 1.0%, with oral steroid prescriptions from 35 to 30%, and patients with a composite exacerbation outcome defined as having one or more of ED visits, hospitalizations, and patients with oral steroid prescriptions will decrease from 40 to 35%. Results of asthma outcomes for each practice will be presented to the practice at the 12-month post kick-off visit along with comparison aggregate data from the other implementation and control sites.

Safety oversight will be under the direction of a Data and Safety Monitoring Board. The DSMB will provide its input to National Institutes of Health staff.

Stakeholder engagement will prove valuable for identifying and addressing barriers to implementation, dissemination, and incorporation of results into practice. Ineffective communication [110, 111], low cultural competence [112], and lack of humility [113] are key barriers when working with vulnerable patients with low income, children, and minority groups [114–117].

### Anticipated barriers and potential solutions

Problems with practice recruitment: As previously mentioned (Sub Aim 1.2), we have a strong record of practice collaboration utilizing healthcare system provider champions who support practice recruitment, implementation, and practice-feedback. By using a tailored approach [99], we hope to fully and effectively implement Coach McLungs^SM^ at 21 practices from a designated selection of ~ 75 practices available for recruitment within a large healthcare system. As previously discussed, a flexible implementation approach includes having a 9-month time window for practice recruitment; adapting the intervention for practice staff and provider preferences; other logistics such as rollout details, additional education needs, and adapting individual practice work flow logistics for use of Coach McLungs^SM^.

Problems with low provider engagement: Providers will be offered support with any potential barriers to using Coach McLungs^SM^. Additionally, during practice feedback, average Coach McLungs^SM^ enrollment numbers will be presented along with comparisons of aggregate data from all 21 practices to allow further discussion of practice or provider specific barriers.

Problems with low survey response: Provider surveys are designed using CFIR constructs to ask no more than 10 relevant questions taking ~ 5 min. Based on this approach our ED project obtained 100% return of pre-implementation ED surveys.

In summary, through previous experience with practice implementation across multiple healthcare settings, and extensive work with provider and patient partners and key stakeholders in the design and preparation for this implementation study, we anticipate overcoming barriers by responsively tailoring the implementation of Coach McLungs^SM^ to the needs of providers and culture of each practice. Understanding best practices for implementation and measuring patient-centered outcomes, such as changes in ED visits and hospitalizations, will inform the clinical and health services research communities with a goal to effect change in both healthcare delivery and health outcomes.

## Supplementary Information

**Additional file 1.** PRE-Implementation Consolidated Framework for Implementation Research.

**Additional file 2**. Organizational Readiness for Implementing Change.

**Additional file 3.** POST Implementation Consolidated Framework for Implementation Research 6 months.

**Additional file 4.** POST Implementation Consolidated Framework for Implementation Research 12 months.

**Additional file 5.** POST Implementation Consolidated Framework for Implementation Research 18 months.

**Additional file 6.** Patient Interview Guide.

**Additional file 7.** Provider Interview Guide.

**Additional file 8.** Patient Asthma Knowledge, Satisfaction, Efficacy.

**Additional file 9.** Provider SDM-Q Doc Physician.

## Data Availability

Not applicable.

## Abbreviations

ED: Emergency department
SDM: Shared decision making
IT: Information technology
EMR: Electronic medical record
