# Supplementary material for: Implementation of Coach McLungsSM into primary care using a cluster randomized stepped wedge trial design
Source: BMC Med Inform Decis Mak. 2022 Nov 4;22:285. doi: 10.1186/s12911-022-02030-1 (PMC9636750; doi:10.1186/s12911-022-02030-1)
Supplement: Supplementary file 1 — Additional file 1. PRE-Implementation Consolidated Framework for Implementation Research. [file 12911_2022_2030_MOESM1_ESM.pdf]

PRE-Implementation CFIR

Please complete the survey below.

Thank you!

What is the name of the Atrium Health Primary Care Practice where you work? ________________________

What is your role in the practice?
- ◯ Faculty Physician or Attending Physician
- ◯ Nurse
- ◯ Health Tech
- ◯ Manager or other leadership
- ◯ Resident Physician
- ◯ Advance Care Practitioner
- ◯ Other Staff

If "other staff", please specify your role. ________________________

From your perspective as a member of the primary care team, how big of a problem is uncontrolled pediatric asthma?

Not a problem — Not sure — Big problem

(Place a mark on the scale above)

For the following statements, how would you rate the level of knowledge of your pediatric patients with uncontrolled asthma (or their caregiver if applicable)?

	Very poor	Poor	Average	Good	Excellent
Recognizing asthma signs & symptoms	◯	◯	◯	◯	◯
Asthma medication adherence	◯	◯	◯	◯	◯
Asthma treatment options	◯	◯	◯	◯	◯
What is asthma? (swelling & inflammation, extra mucus, tightening of the airways)	◯	◯	◯	◯	◯
Identifying asthma triggers and avoidance strategies	◯	◯	◯	◯	◯
Asthma inhaler technique	◯	◯	◯	◯	◯

To improve the care for pediatric patients with asthma, should the following be addressed before or during the asthma visit? Please rate your level of agreement with the following statements. (Mark one answer for each line)

	Strongly disagree	Somewhat disagree	Neither disagree or agree	Somewhat agree	Strongly agree
Improve patient education	◯	◯	◯	◯	◯
Improve patient experience	◯	◯	◯	◯	◯

Linkage to primary care	○	○	○	○	○
ACCESS to primary care	○	○	○	○	○
Increase patient self-management	○	○	○	○	○
Decision support for provider and patient	○	○	○	○	○

Are there any other topics that should be addressed to improve care for pediatric patients visiting the primary care with asthma? __________________________________

Please rate your level of agreement with the following statements. (Mark one answer for each line)

	Strongly disagree	Somewhat disagree	Neither disagree or agree	Somewhat agree	Strongly agree
I am willing to use new and different types of programs developed by researchers	○	○	○	○	○
I would try a new program even if it is very different from what I am used to doing	○	○	○	○	○
Leadership strongly supports change efforts in primary care	○	○	○	○	○
Primary care leadership makes sure that we have the time and space necessary to discuss changes to improve care	○	○	○	○	○
In general, when there is agreement that change needs to happen in primary care, we have the necessary support in terms of training	○	○	○	○	○
In general, when there is agreement that change needs to happen in primary care, we have the necessary support in terms of staffing	○	○	○	○	○
People in our primary care practice actively seek new ways to improve how we do things	○	○	○	○	○
