# Supplementary material for: Implementation of Coach McLungsSM into primary care using a cluster randomized stepped wedge trial design
Source: BMC Med Inform Decis Mak. 2022 Nov 4;22:285. doi: 10.1186/s12911-022-02030-1 (PMC9636750; doi:10.1186/s12911-022-02030-1)
Supplement: Supplementary file 2 — Additional file 2. Organizational Readiness for Implementing Change. [file 12911_2022_2030_MOESM2_ESM.pdf]

Organizational Readiness for Implementing Change (ORIC)

We are interested in your opinions about your clinic's collective readiness to implement Coach McLungs. Collective readiness refers to the willingness and ability of the all the members in the clinic who are involved in the use of Coach McLungs. Therefore, please consider this entire group of individuals in your clinic (i.e., "We"), not just yourself, when selecting your responses. Your responses will remain anonymous.

Please rate your level of agreement with the following statements. (Mark one answer for each line)

		Disagree	Somewhat disagree	Neither agree nor disagree	Somewhat agree	Agree
1)	We want to implement Coach McLungs.	○	○	○	○	○
2)	We feel confident that we can handle the challenges that might arise in implementing Coach McLungs.	○	○	○	○	○
3)	We feel confident that we can keep track of progress in implementing Coach McLungs.	○	○	○	○	○
4)	We are determined to implement Coach McLungs.	○	○	○	○	○
5)	We feel confident that we can coordinate tasks so that implementation goes smoothly.	○	○	○	○	○
6)	We feel confident that the organization can support us as we adjust to Coach McLungs.	○	○	○	○	○
7)	We are committed to implementing Coach McLungs.	○	○	○	○	○
8)	We feel confident that we can manage the politics of implementing Coach McLungs.	○	○	○	○	○
9)	We will do whatever it takes to implement Coach McLungs.	○	○	○	○	○
