# Supplementary material for: Implementation of Coach McLungsSM into primary care using a cluster randomized stepped wedge trial design
Source: BMC Med Inform Decis Mak. 2022 Nov 4;22:285. doi: 10.1186/s12911-022-02030-1 (PMC9636750; doi:10.1186/s12911-022-02030-1)
Supplement: Supplementary file 5 — Additional file 5. POST Implementation Consolidated Framework for Implementation Research 18 months. [file 12911_2022_2030_MOESM5_ESM.pdf]

POST Implementation CFIR 18 months

Please complete the survey below.

Thank you!

____

What is the name of the Atrium Health Primary Care Practice where you work?	__________________________________
What is your role in the practice?	◯ Faculty Physician or Attending Physician<br>◯ Nurse<br>◯ Health Tech<br>◯ Manager or other leadership<br>◯ Resident Physician<br>◯ Advanced Practice Providers<br>◯ Other Staff
If other staff, please specify your role.	__________________________________
Did you complete the previous 6- and/or 12-month post-implementation survey(s) as part of Coach McLungs?	◯ Yes<br>◯ No
From your perspective as a member of the primary care team, how big of a problem is uncontrolled pediatric asthma?	Not a problem — Not sure — Big problem<br>*(Place a mark on the scale above)*

For the following statements, how would you rate the level of knowledge of your pediatric patients with uncontrolled asthma (or their caregiver if applicable)?

	Very poor	Poor	Average	Good	Excellent
Recognizing asthma signs & symptoms	◯	◯	◯	◯	◯
Asthma medication adherence	◯	◯	◯	◯	◯
Asthma treatment options	◯	◯	◯	◯	◯
What is Asthma? (swelling & inflammation, extra mucus, tightening of the airways)	◯	◯	◯	◯	◯
Identifying triggers & avoidance strategies	◯	◯	◯	◯	◯
Asthma inhaler technique	◯	◯	◯	◯	◯

Please rate your level of agreement with the following statements. (Mark one answer for each line)

	Strongly disagree	Somewhat disagree	Neither disagree or agree	Somewhat agree	Strongly agree
Leadership strongly supports change efforts in primary care	◯	◯	◯	◯	◯
Primary care leadership makes sure we have the time and space necessary to discuss changes to improve asthma care	◯	◯	◯	◯	◯

Are you familiar with the Atrium Health Asthma Coach, "Coach McLungs?" ◯ Yes ◯ No

In thinking about your experience with the Asthma Coach, please rate your level of agreement with the following statements. (Mark one answer for each line)

I believe Coach McLungs...

	Strongly disagree	Somewhat disagree	Neither disagree or agree	Somewhat agree	Strongly agree
improves patient education	◯	◯	◯	◯	◯
improves patient experience	◯	◯	◯	◯	◯
improves linkage to primary care	◯	◯	◯	◯	◯
increases patient self-management	◯	◯	◯	◯	◯
provides decision support for provider and patient	◯	◯	◯	◯	◯
is engaging and fun for the patient	◯	◯	◯	◯	◯
helps fill gaps in asthma care	◯	◯	◯	◯	◯

Please rate your level of agreement with the following statements. (Mark one answer for each line)

	Strongly disagree	Somewhat disagree	Neither disagree or agree	Somewhat agree	Strongly agree
Our primary care staff is getting the support we need to implement Coach McLungs	◯	◯	◯	◯	◯

Using Coach McLungs is better than other education materials to improve asthma education and treatment planning	◯	◯	◯	◯	◯
I felt I had enough training to use Coach McLungs correctly	◯	◯	◯	◯	◯
I think that using Coach McLungs to improve asthma care fits well with the way I like to work	◯	◯	◯	◯	◯
Managers actively support implementation of Coach McLungs	◯	◯	◯	◯	◯
Overall, I believe it was easy to implement Coach McLungs at our primary care practice	◯	◯	◯	◯	◯

Are there any other thoughts you would like to share about Coach McLungs?

____

Would you be willing to take part in a virtual key informant interview (over the phone) to tell us more about the implementation of Coach McLungs at your practice?

◯ Yes
◯ No

Please write your first and last name, and the best telephone number where we can reach you.

An Atrium Health Research Coordinator will be in touch to discuss scheduling the key informant interview on a date and time most convenient with your schedule.

____
