# Supplementary material for: Implementation of Coach McLungsSM into primary care using a cluster randomized stepped wedge trial design
Source: BMC Med Inform Decis Mak. 2022 Nov 4;22:285. doi: 10.1186/s12911-022-02030-1 (PMC9636750; doi:10.1186/s12911-022-02030-1)
Supplement: Supplementary file 6 — Additional file 6. Patient Interview Guide. [file 12911_2022_2030_MOESM6_ESM.pdf]

Introduction:

Thank you for agreeing to speak with me today. You were invited to participate in this interview because at a previous doctor's visit you/your child used Coach McLungs, a new web-based asthma app. The goal of this program is to provide patients, parents, and caregivers asthma decision-making support. With your permission, I would like to ask you some questions about your experience with Coach McLungs. I would like to see how the program fit at your/your child's doctor's office. There are no right or wrong answers to any of the following questions, only what feels right to you. Is there anything you would like to ask me before we begin?

I would like to start by asking about your/your child's history with asthma.

	Interview Question	CFIR
**1.**	**How many years have you/your child had asthma?**	*History*
**2.**	**Have you/your child ever gone to the Emergency Room (ER) for help with asthma?**<br><br>○ ***If YES*** *- How many times in the past year have you/your child been to the Emergency Room for asthma?*	*History*
**3.**	**Can you tell me where you/your child used Coach McLungs?**<br>[pause for answer] Read the following probes, if needed:<br>a. On an iPad in the waiting room<br>b. On an iPad in the exam room<br>c. On a computer in the exam room<br>d. On a computer at home before going to see my doctor<br>e. On an iPad or tablet at home before going to see my doctor<br>f. On a smartphone at home before going to see my doctor<br>i. If YES - Do you have access to a computer or iPad?<br><br>g. If none of above options - Can you tell me where you/your child used Coach McLungs?<br><br>Probe:<br>○ **Where would you like to use Coach McLungs?**	*D: Process*<br>*C: Executing*
**4.**	**Do you feel you got help from staff at your doctor's office to use Coach McLungs?**<br><br>○ ***If YES* - Can you tell me how the staff helped you?**<br>○ ***If NO* - What type of support would've been helpful?**	*D: Inner Setting*<br>*C: Implementation Climate*<br>*(Fernandez)*
**5.**	**Based on other education materials you've received from your doctor, how did Coach McLungs compare to help you with your asthma?**	*D: Innovation Characteristics*<br>*C: Relative Advantage*<br>*(Kegler)*

	Probes:<br>○ **What did you like or dislike about Coach McLungs?**<br>○ **Did Coach McLungs help you/your child learn about asthma triggers and ways to avoid them?**<br>○ **Did you learn about the difference between controller and rescue medications?**<br>○ **Did you talk about a treatment plan with your doctor?**<br>○ **Did Coach McLungs help you/your child talk about concerns and choices with the doctor?**<br>○ **Did Coach McLungs help you/you child review asthma medications with doctor?**	
**6.**	**Did Coach McLungs make sense to you or were there parts you found hard to understand?**<br><br>Probes:<br>○ ***If YES* - what parts were confusing?**<br>○ ***If NO* - what parts of Coach McLungs worked well?**	*D: Characteristics of Individuals*<br>*C: Knowledge & Beliefs of the Innovation*<br>*(Kegler)*
**7.**	**Do you think using Coach McLungs to improve asthma fits well into a doctor's visit?**<br><br>Probes:<br>○ **Do you think Coach McLungs was useful?**<br>○ **Was the time it took to use the app, did it add value to your visit?**<br>○ ***If YES* - what did you like about using Coach McLungs at your doctor's visit?**<br>○ ***If NO* - what did you not like about using Coach McLungs at your doctor's visit?**	*D: Innovation Characteristics*<br>*C: Compatibility*<br>*(Kegler)*
**8.**	**What do you think of the playbook summary, or paper handout, you got after using Coach McLungs?**<br><br>○ Probes:<br>○ **Was it helpful to you?**<br>○ **Was the playbook/paper handout easy to understand?**<br>○ **Did you review the playbook/paper handout [with your child's] doctor during the visit? Or, at home?**<br>○ **Were all your asthma questions answered during the visit?**	*D: Characteristics of Individuals*<br>*C: Knowledge & Beliefs of the innovation*<br>*(Kegler)*
**9.**	**Overall, how easy, or hard was it for you/your child to use Coach McLungs at your doctor's office [at home]?**<br><br>Probe:	*D: Innovation Characteristics*<br>*C: Complexity*<br>*(Kegler)*

	○ ***What made it easy? What made it hard?***	
**10.**	**Are there any other ways Coach McLungs helped you/your child?**	*D: Outer Setting*<br>*C: Needs & Resources of Those Served by the Organization*
**11.**	**Those are all the questions I have. Is there anything else you would like to share about Coach McLungs?**	
