# Supplementary material for: Implementation of Coach McLungsSM into primary care using a cluster randomized stepped wedge trial design
Source: BMC Med Inform Decis Mak. 2022 Nov 4;22:285. doi: 10.1186/s12911-022-02030-1 (PMC9636750; doi:10.1186/s12911-022-02030-1)
Supplement: Supplementary file 7 — Additional file 7. Provider Interview Guide. [file 12911_2022_2030_MOESM7_ESM.pdf]

Introduction:

Thank you for agreeing to speak with me today on the implementation of Coach McLungs. You were invited to participate in this interview because you are a provider/staff at [Practice Name] using Coach McLungs, a shared-decision-making web-based asthma app. The aim is to evaluate implementation barriers and facilitators to tailor Coach McLungs for use in a number of primary care settings. We are interested in hearing your experience and opinions. There are no right or wrong answers, only what feels right to you. Do you have questions before we begin?

	Interview Question	CFIR
**1.**	**Can you start by telling me about your role, and walk me through how Coach McLungs was implemented at [Practice Name]?**<br><br>• Probes:<br>○ ***How did you first hear about Coach McLungs?***<br>*D: Inner Setting, C: Networks and Communication*<br>*D: Inner Setting, C: Access to Knowledge & Information*<br>○ **Did your team have established goals for implementing Coach McLungs?**<br>*D: Inner Setting, C: Goals & Feedback*	*D: Process*<br>*C: Reflecting & Evaluating*
**2.**	**Do you think Coach McLungs was successfully implemented at your practice?**<br><br>○ Probes:<br>○ ***What do you think went well with implementation?***<br>*D: Process, C: Executing*<br>○ ***What are some of the things that didn't go well?***<br>*D: Process, C: Executing*<br>○ ***Do you think the training was useful and/or sufficient for using Coach McLungs at your practice?***	*D: Characteristics of Individuals*<br>*C: Knowledge & Beliefs about the Innovation*
**3.**	**Do you feel confident using Coach McLungs with patients? Why or why not?**<br><br>Probe:<br>○ ***Was the time it took to use the app with a patient, did it add value?***	*D: Characteristics of Individuals*<br>*C: Self-Efficacy*<br>*D: Characteristics of Individuals*<br>*C: Individual Stage of Change*<br>*D: Innovation Characteristics*<br>*C: Complexity*
**4.**	**What level of involvement does practice leadership have with implementation of Coach McLungs?**<br><br>• Probes:<br>○ ***Who do you see as leadership at [Practice Name]?***<br>*D: Characteristics of Individuals, C: Individual identification with Organization*	*D: Inner Setting*<br>*C: Leadership Engagement*

	D: Process, C: Opinion Leaders<br>○ ***Are there specific champions within your practice that are often early adopters of new processes or innovations? How little or great of an influence do they have on the team?***<br>*D: Process, C: Champions*	
**5.**	**Do you feel you have enough resources to implement Coach McLungs at your practice, such as space, iPads, clinic staff?**<br>○ Probe:<br>○ ***Are there other resources you feel would help to the implementation of Coach McLungs?***<br>*D: Process, C: Reflecting and Evaluating*	*D: Inner Setting*<br>*C: Available Resources*
**6.**	**How well do you think Coach McLungs fits with existing work processes at your practice?**<br>• Probe:<br>○ ***What is the level of receptivity at your practice towards the implementation of Coach McLungs?***<br>*D: Inner Setting, C: Implementation Climate*<br>○ ***Are there things about the program that you like or find helpful for patients? Such as…***<br>▪ ***Asthma medications discussions – step up/step down***<br>▪ ***Collecting history***<br>▪ ***Decision support for provider and patient/family***<br>▪ ***Patient Education***<br>*D: Innovation Characteristics, C: Relative Advantage*<br>*D: Outer Setting, C: Needs & Resources of Those Served by the Organization*<br>○ ***Describe your ideal process for using Coach McLungs.***<br>*D: Process, C: Planning*	*D: Inner Setting*<br>*C: Compatibility*
**7.**	**How are patients at your practice reacting to Coach McLungs?**<br>○ Probe:<br>○ ***What barriers do pediatric patients face when using Coach McLungs at your practice?***<br>*D: Process, C: Innovation Participants*	*D: Outer Setting*<br>*C: Needs & Resources of Those Served by the Organization*
**8.**	**What is your overall opinion of Coach McLungs?**	*D: Characteristics of Individuals*<br>*C: Knowledge and Beliefs of the Innovation*
**9.**	**This concludes all of my questions. Is there anything else you would like to share about the implementation of Coach McLungs at your practice?**	
