# Supplementary material for: Implementation of Coach McLungsSM into primary care using a cluster randomized stepped wedge trial design
Source: BMC Med Inform Decis Mak. 2022 Nov 4;22:285. doi: 10.1186/s12911-022-02030-1 (PMC9636750; doi:10.1186/s12911-022-02030-1)
Supplement: Supplementary file 8 — Additional file 8. Patient Asthma Knowledge, Satisfaction, Efficacy. [file 12911_2022_2030_MOESM8_ESM.pdf]

Patient Asthma Knowledge, Satisfaction, Efficacy (Built In)

Please complete the survey below.

Thank you!

____

First Name: [pt_first_name]
Last Name: [pt_last_name]
Birthdate: [pt_birthdate]
MRN: [pt_mrn]
Phone Number: [pt_phone_number]

Fill in the blank: I think Coach McLungs was ____________________

- ◯ Extremely helpful
- ◯ Very helpful
- ◯ Somewhat helpful
- ◯ Not very helpful
- ◯ Not helpful at all

After talking to Coach McLungs, would you say.... "I have a good understanding of how to control asthma."

- ◯ Yes, definitely
- ◯ Yes, a little
- ◯ Maybe
- ◯ No, not really
- ◯ No, definitely not

Would you say..... "I know what to talk to the doctor about today?"

- ◯ Yes, definitely
- ◯ Yes, a little
- ◯ Maybe
- ◯ No, not really
- ◯ No, definitely not

Do you have any additional comments - good or bad?

____

Please identify participants willing to take part in a key informant interview by asking the following questions:

Would you be interested in taking part in a phone interview to tell us more about your experience using Coach McLungs at your doctor's office?

- ◯ yes
- ◯ no

Please add your first name and telephone number.

An Atrium Health teammate will call you to talk about the phone interview in more detail. Thank you for your interest.

____
