# Supplementary material for: Implementation of Coach McLungsSM into primary care using a cluster randomized stepped wedge trial design
Source: BMC Med Inform Decis Mak. 2022 Nov 4;22:285. doi: 10.1186/s12911-022-02030-1 (PMC9636750; doi:10.1186/s12911-022-02030-1)
Supplement: Supplementary file 9 — Additional file 9. Provider SDM-Q Doc Physician. [file 12911_2022_2030_MOESM9_ESM.pdf]

Provider SDM-Q Doc Physician (12 month)

Please complete the survey below.

Thank you!

First Name: [pt_first_name]
Last Name: [pt_last_name]
Birthdate: [pt_birthdate]
MRN: [pt_mrn]
Phone Number: [pt_phone_number]

1) Please indicate which health complaint/problem/illness the consultation was about: ______________________________

2) Please indicate which decision was made: ______________________________

Nine statements related to the decision-making in the above mentioned consultation are listed below. For each statement, please indicate how much you agree or disagree.

	completely disagree	strongly disagree	somewhat disagree	somewhat agree	strongly agree	completely agree
3) I made clear to my patient that a decision needs to be made.	○	○	○	○	○	○
4) I wanted to know exactly from my patient how he/she wants to be involved in making the decision.	○	○	○	○	○	○
5) I told my patient that there are different options for treating his/her medical condition.	○	○	○	○	○	○
6) I precisely explained the advantages and disadvantages of the treatment options to my patient.	○	○	○	○	○	○
7) I helped my patient understand all the information.	○	○	○	○	○	○
8) I asked my patient which treatment option he/she prefers.	○	○	○	○	○	○
9) My patient and I thoroughly weighed the different treatment options.	○	○	○	○	○	○
10) My patient and I selected a treatment option together.	○	○	○	○	○	○
11) My patient and I reached an agreement on how to proceed.	○	○	○	○	○	○
